# Selected research articles from the 2016 International Workshop on Computational Network Biology: Modeling, Analysis, and Control (CNB-MAC)

**DOI:** 10.1186/s12859-017-1521-3

**Published:** 2017-03-22

**Authors:** Byung-Jun Yoon, Xiaoning Qian, Tamer Kahveci

**Affiliations:** 1 Department of Electrical and Computer Engineering, Texas A&M University, College Station, TX 77843 USA; 2 TEES-AgriLife Center for Bioinformatics and Genomic Systems Engineering (CBGSE), College Station, TX 77845 USA; 3 Department of Computer and Information Science and Engineering, University of Florida, Gainesville, FL 32611-6125 USA

## Introduction

The Third International Workshop on Computational Network Biology: Modeling, Analysis, and Control (CNB-MAC 2016) was held in Seattle, Washington on October 2, 2016. As in previous years, the workshop was organized in conjunction with the ACM Conference on Bioinformatics, Computational Biology, and Health Informatics (ACM-BCB), the flagship conference of the ACM SIGBio. This workshop aims to provide an international scientific forum for presenting recent advances in computational network biology that involve modeling, analysis, and control of biological systems and system-oriented analysis of large-scale OMICS data.

CNB-MAC 2016 was co-chaired by Drs. Byung-Jun Yoon, Xiaoning Qian, and Tamer Kahveci. The workshop featured a keynote speech by Dr. Su-In Lee from the Department of Computer Science & Engineering and the Department of Genome Sciences at the University of Washington entitled “Mining Big Data for Molecular Marker Identification.” The workshop also featured presentations of 12 original research papers [1–12], 3 posters, and 2 highlight presentations of recently published journal papers [13, 14].

## Research papers presented at CNB-MAC 2016

Twelve original research papers [1–12] have been selected for presentation at CNB-MAC 2016.

Ahsen et al. [1] proposed a novel sparse feature selection algorithm for classification in a small sample setting, where the number of potential features is significantly larger than the sample size. The algorithm was used to build a classifier for predicting the metastasis of endometrial cancer using the expression signature of 18 microRNAs. Evaluation results showed that the designed classifier could accurately predict the metastatic status in endometrial cancer patients.

Accurate inference of Microbial Interaction Networks (MIN) is an important step toward better understanding the dynamics that underlie bacterial ecosystems. Alshawaqfeh et al. [2] proposed a novel algorithm for MIN inference, called the SgLV-EKF. The algorithm represents the MIN as a nonlinear stochastic system using the generalized Lotka-Volterra (gLV) model. This model is inferred by applying the extended Kalman filter (EKF). Performance assessment on real and synthetic datasets demonstrated the effectiveness of SgLV-EKF in predicting MINs and tracking their dynamics, where the algorithm was shown to outperform existing methods.

Arshad and Datta [3] investigated the problem of designing effective combinatorial therapies for prostate cancer patients. In their work, they adopted a Boolean circuit model to represent the gene regulatory network responsible for the development and progression of prostate cancer. The constructed circuit model was then used to design combinatorial therapies that are expected to most effectively counteract the abnormalities in the gene regulatory network. The proposed framework has the potential to provide the scientific ground for designing targeted therapies for prostate cancer patients.

Matlock et al. [4] considered the problem of designing personalized targeted cancer therapies. They formulated the problem as an optimization problem, where the goal is to maximize the drug efficacy on tumor cells while minimizing the toxicity of the drug over normal cells. An accelerated lexicographic search algorithm was designed to find the optimal solution, which takes advantage of the properties of the probabilistic target inhibition map (PTIM) – that models tumor proliferation – to considerably cut down the search space. The authors demonstrated that their algorithm could provide a computational framework for designing effective combination drugs while keeping their overall toxicity at an acceptable level.

Aadi et al. [5] developed a novel framework for time-frequency analysis of genomic sequences. Using the notion of interpretive signal processing, the authors extended conventional time-frequency transforms for non-numerical sequences, resulting in a machinery that can naturally handle symbolic sequences such as genomic sequences. Based on synthetic and real DNA sequences, the authors showed how the proposed method could be used to detect periodicities and tandem repeats in the genome.

Sonmez and Can [6] presented a method for analyzing the graphlet signatures in integrated genome-scale networks. They analyzed the interactions between human proteins – including physical, regulatory, and metabolic interactions – obtained from the Pathway Commons database, and counted the sub-graph patterns in the network to identify overrepresented graphlets that are statistically significant. Sonmez and Can showed that the graphlet signature vector, which contains the graphlet counts, could provide an effective means of system level analysis of tissue/disease specific networks.

Recent advances in next-generation sequencing technologies have made RNA-Seq increasingly popular for obtaining trancriptomic profiles. When RNA-Seq data contain mixed signals from various cell-types and/or tissues, deconvolution techniques could be used to pick out the signal that belongs to a specific cell-type or tissue. Noting that most deconvolution algorithms make linearity assumptions, Jin and Liu [7] made comprehensive assessment of various RNA-Seq quantification methods to find out which method yields the optimum linear space for such deconvolution analysis. Their analysis showed that directly using the count data leads to poor estimation results, while TPM (transcripts per million) values estimated from Salmon [15] and Kallisto [16] were optimal for deconvolution studies.

The reaction diffusion master equation (RDME) is widely used for stochastic simulation of spatiotemporal biological systems, providing a coarse-grained framework that is well suited for large-scale simulation of reaction–diffusion systems. In [8], Chen et al. focused on stochastic modeling of reaction–diffusion systems with reaction rate laws given by Hill functions. They showed that the RDME framework faces critical simulation defects when the discretization size gets smaller than a certain microscopic limit, in which case the switch-like Hill dynamics becomes linear to the input and the discretization size. Based on this observation, Chen et al. proposed methods that could be used to prevent this problem, and thereby correctly simulate Hill function dynamics in the microscopic RDME system.

Rare diseases affecting only a small fraction of the population are referred to as orphan diseases, most of which are genetic in origin. In [9], Liu et al. proposed a novel method for detecting potential causative genes through the analysis of protein-protein (PPI) networks. The proposed method, called DIGNiFI (disease causing gene finder), prioritizes disease gene candidates based on the assumption that genes with high topological similarity tend to be associated with phenotypically similar disorders. The topological similarity between two genes is computed by considering their common direct neighbors and performing local random walks, and the results are further improved by utilizing gene ontology (GO) annotations. DIGNiFI was applied to the prediction of novel candidate genes that might be responsible for four inherited retinal dystrophies, which confirmed that the top predictions made by DIGNiFI were consistent with reports in existing literature and databases.

It is well known that the risk of developing alcohol use disorder is affected by genetics as well as various environmental factors. To investigate the potential interactions among genetic and environmental factors and their effect on alcoholism, Zollanvari and Alterovitz [10] analyzed a network of SNP by SNP by Environment interactions. Assessment of the SNPxSNPxE network constructed from the data for 3,776 individuals led to the prediction of genes, SNP-SNP interactions, and SNP-E interactions that are likely to be associated with alcoholism, and it also detected pathways that appear to be linked to alcoholism.

Network querying aims to detect subnetwork regions in a target network that are similar to a given query network, in terms of topology and composition, and it can provide an effective computational means of predicting orthologous pathways in different species. In [11], Jeong and Yoon proposed a novel algorithm for querying networks, called SEQUOIA, that can improve the biological significance of the network querying results. SEQUOIA compares the query and the target networks using a context-sensitive random walk (CSRW) model, thereby predicting the node-to-node correspondence between the networks. The resulting CSRW scores are used to detect highly similar subnetworks in the target, which are then subsequently extended by minimizing the network conductance. Evaluation based on real PPI networks and known molecular complexes showed that the proposed algorithm outperforms other state-of-the-art querying methods.

Wang and Qian [12] presented a novel algorithm for protein complex prediction, called FLCD (Finding Low-Conductance sets with Dense interactions). FLCD is a two-step algorithm. First step identifies potential low-conductance sets in a PPI network using a personalized PageRank vector and then solving a mixed integer programming (MIP) problem. Second step searches within these sets for densely connected network modules by solving another MIP problem. Experiments based on large-scale yeast PPI networks demonstrated that protein complexes predicted by FLCD have better correspondence to the yeast protein complex gold standards compared to predictions made by other state-of-the-art algorithms.
